# Short QRS Duration After His-Purkinje Conduction System Pacing Predicts Left Ventricular Complete Reverse Remodeling in Patients With True Left Bundle Branch Block and Heart Failure

**DOI:** 10.3389/fcvm.2022.824194

**Published:** 2022-05-06

**Authors:** Xu-Min Guan, Dan-Na Li, Fu-Lu Zhao, Yan-Ni Zhao, Yi-Heng Yang, Bai-Ling Dai, Shi-Yu Dai, Lian-Jun Gao, Yun-Long Xia, Ying-Xue Dong

**Affiliations:** Department of Cardiology, The First Affiliated Hospital of Dalian Medical University, Dalian, China

**Keywords:** His-Purkinje conduction system pacing, left bundle branch block, heart failure, QRS duration, predictors

## Abstract

**Objective:**

This study aimed to explore the outcomes of His-Purkinje conduction system pacing (HPCSP) and to screen the predictors of left ventricular (LV) complete reverse remodeling in patients with true left bundle branch block (LBBB) and heart failure with reduced ejection fraction (HFrEF).

**Methods:**

Patients who underwent HPCSP for true LBBB and HFrEF from April 2018 to August 2020 were consecutively enrolled. All participants were followed up for at least 1 year. Thrombosis, infection, lead dislodgement, perforation, and other complications were observed after HPCSP. Clinical data, including echocardiographic parameters, electrocardiogram measurements, and cardiac function, were assessed before and after the procedure.

**Results:**

A total of 46 patients were enrolled. HPCSP was successfully deployed in 42 cases (91.30%), which included 37 cases with His bundle pacing (HBP) and 5 cases with left bundle branch pacing (LBBP). The QRS duration decreased significantly (169.88 ± 19.17 ms vs. 113.67 ± 20.68 ms, *P* < 0.001). Left ventricular end-systolic volume (LVESV) (167.67 ± 73.20 ml vs. 85.97 ± 62.24 ml, *P* < 0.001), left ventricular end-diastolic diameter (LVEDD) (63.57 ± 8.19 mm vs. 55.46 ± 9.63 mm, *P* = 0.003) and left ventricular ejection fraction (LVEF) (26.52 ± 5.60% vs. 41.86 ± 11.56%, *P* < 0.001) improved dramatically. Complete reverse remodeling of the LV with normalized LVEF and LVEDD was found in nearly half of the patients (45.24%). A short QRS duration after HPCSP was a strong predictor of normalized LVEF and LVEDD (*P* < 0.001). The thresholds increased markedly in two patients approximately 6 months after HBP. No patients died during the total follow-up period of 20.07 ± 6.45 months.

**Conclusion:**

Complete reverse remodeling of the LV could be found in nearly half of the patients with HFrEF and true LBBB after HPCSP, and the short QRS duration after HPCSP was a strong predictor.

## Introduction

Approximately 30% of patients with heart failure and left ventricular (LV) desynchronization showed no response to cardiac resynchronization therapy (CRT) *via* conventional biventricular pacing (BiVP) (1, 2). A greater response to BiVP was found in patients with true left bundle branch block (LBBB) (3).

Several studies have illustrated that His-Purkinje conduction system pacing (HPCSP), including His bundle pacing (HBP) and left bundle branch pacing (LBBP), could be a better option for CRT (4–8). Singh et al. demonstrated that normalized LVEF was found in 71.43% of patients with LBBB-induced cardiomyopathy after HPCSP (9). How can the proportion of LV complete reverse remodeling with normalized LVEF and LV end-diastolic diameter (LVEDD) be maximized? Obviously, the predictors are still unclear. This study aimed to evaluate the efficacy of the HPCSP and explore the predictors of LV complete reverse remodeling in patients with true LBBB and heart failure with reduced ejection fraction (HFrEF).

## Materials and Methods

### Patient Enrollment and Follow-Up

Patients with true LBBB and HFrEF who underwent HPCSP from April 2018 to August 2020 were consecutively enrolled in our center. The exclusion criteria were recent myocardial infarction or cardiac surgery (<3 months). All patients consented to their treatment, which was approved by the hospital ethics board. LBBP would be the alternative therapy for those patients whose first choice of HBP failed, and BiVP would be the rescue therapy if HPCSP failed. All patients received guideline-directed medical therapy for at least 3 months before implantation.

Regular follow-up was conducted 1, 3, 6, 12, 18, and 24 months after the operation. During the follow-up, the 12-lead electrocardiogram (ECG), echocardiography, postoperative complications, and pacemaker parameters were monitored. The events of thrombosis, infection, lead dislodgement, perforation, stroke, rehospitalization due to heart failure, or death were recorded.

The left ventricular end-systolic volume (LVESV), LVEDD, and left atrial diameter (LAD) were measured following the guidelines of the American Society of Echocardiography. LVEF was measured using the biplane Simpson’s method, and the maximum mitral regurgitation (MR) and tricuspid regurgitation (TR) were measured by the vena contracta width with color-flow Doppler.

### Criteria and Definition

True LBBB was defined as QRS duration > 140 ms in men (>130 ms in women) and the presence of at least 2 mid-QRS notches or slurs in leads I, aVL, V_1_, V_2_, V_5_, and V_6_ (10). An LVEF higher than 50% and an LVEDD less than 50 mm were considered LV complete reverse remodeling.

His bundle pacing usually had two independent capture thresholds, including a His-bundle capture threshold and an LBBB correcting threshold in those patients. An abrupt decrease in the Stim-LV active time (LVAT) of more than 10 ms and the morphologies of Qr, qR, or rSR’ in lead V_1_ were the simple criteria for left bundle branch capture.

### Implantation Procedure and Device Programme

The HBP and LBBP were performed using the Select Secure pacing lead (Model 3830, 69 cm, Medtronic Inc., Minneapolis, MN, United States) and a fixed-curve sheath (C315 HIS, Medtronic Inc., Dublin, Ireland). His bundle electrograms were mapped in a unipolar configuration and recorded in the system (Prucka Cardiolab, GE Healthcare, Waukesha, WI, United States). As described in our previous publications, LBBB correcting thresholds lower than 3.0 V/0.4 ms were accepted (11).

The LBBP was further performed when HBP failed to correct LBBB or when the corrected threshold was above 3.0 V/0.4 ms. The sheath and lead were advanced approximately 1–2 cm from the His bundle region. The unipolar-tip paced QRS configuration and pacing impedance were monitored along with the measurement of peak LV activation times in lead V_5_ for LBBP. All patients accepted a CRT defibrillator (D) or CRT pacemaker (P) device according to the guidelines. The leads were then connected to the left atrium (LA), right ventricle (RV), and LV ports. The LV-RV delay was programmed to ensure the shortest QRS duration. The 3,830 lead was connected to the LV port, and the longer interventricular delay was programmed to ensure ventricular activation *via* conduction system pacing.

If HPCSP was unsuccessful, an LV lead was implanted *via* the traditional coronary venous approach. The LV lead was positioned with a standard technique in the lateral or posterolateral LV vein on patients with BiVP if possible. The RV lead was implanted in the right ventricular septum.

### Statistical Analysis

Statistical analyses were performed using SPSS 23.0. Continuous variables were expressed as the mean ± SD or median and were compared with independent two-samples, paired *t*-test, or Wilcoxon test. Categorical variables were expressed as numbers (%) and were compared using the Fisher’s exact test. Univariate and multivariate analyses were performed using logistic regression to determine the independent predictors of LV complete reverse remodeling after HPCSP. The optimal cutoff of QRS duration was shown on the receiver operator characteristic (ROC) curve with the maximized sensitivity and specificity. *P* < 0.05 (two-tailed) was considered to be statistically significant.

## Results

### Baseline Patient Characteristics and Clinical Events

A total of 46 patients were enrolled in this study. The HPCSP was successfully deployed in 42 cases (91.30%), which included 37 cases (80.43%) with HBP and 5 cases (10.87%) with LBBP, and the other 4 patients for whom HPCSP failed accepted BiVP. All patients were implanted with CRT defibrillator (D) (30, 65.22%) or CRT pacemaker (P) devices. All patients were followed up for at least 12 months, and the follow-up duration was 20.07 ± 6.45 months. The LBBB was corrected in all 42 patients after HBCSP with a correcting threshold of 2.13 ± 0.65 V/0.4 ms, and the His-bundle capture threshold was 1.71 ± 0.87.

The baseline characteristics of the patients are shown in Table 1. The average age of the patients was 70.21 ± 9.20 years, the average LVEF value was 26.52 ± 5.60%, and the average QRS duration was 169.88 ± 19.17 ms. During the follow-up, one patient was rehospitalized due to heart failure, and no patients died. Complications such as thrombosis, infection, lead dislodgement, perforation, and stroke were not found in any of the patients. The thresholds increased markedly (3.0 V/1.0 ms) in two patients approximately 6 months after HBP, and then the thresholds decreased to 1.5 V/0.4 ms after resetting the lead.

**TABLE 1:** Baseline characteristics of patients.

	All patients (*n* = 42)
Male (n,%)	22(52.38)
Age (years)	70.21 ± 9.20
Course of heart failure (years)	5.24 ± 3.21
NYHA classification (level)	3.31 ± 0.60
LVEF (%)	26.52 ± 5.60
HBP (n,%)	37(88.10%)
BMI (kg/m2)	25.09 ± 3.47
BNP (ng/L)	438.00(222.50, 1287.50)
Crea (μ mol/L)	72.00(60.00, 89.25)
QRS duration (ms)	169.88 ± 19.17
QRS duration after HPCSP (ms)	113.67 ± 20.68
**MR grade**	
Mild (n,%)	12(28.6)
Moderate (n,%)	25(59.5)
Severe (n,%)	5(11.9)
**TR grade**	
Mild (n,%)	7(16.7)
Moderate (n,%)	23(54.8)
Severe (n,%)	12(28.6)
LVESV (ml)	167.67 ± 73.20
LVEDD (mm)	63.57 ± 8.19
LAD (mm)	44.59 ± 4.12
Diabetes mellitus (n,%)	12(28.57)
Hypertension (n,%)	21(50.00)
Chronic kidney disease (n,%)	2(4.76)
Coronary heart disease (n,%)	13(31.0)
Ventricular tachycardia/fibrillation (n,%)	8(19.0)
Atrial fibrillation (n,%)	8(19.0)
β -blockers (n,%)	39(92.9)
ARNI/ACEI/ARB	40(95.2)
Diuretics (n,%)	40(95.2)
Spirolactone (n,%)	39(92.9)
Statins (n,%)	26(61.9)
Aspirin (n,%)	10(23.8)
Nitrates (n,%)	17(40.5)

*NYHA, New York Heart Association; LVEF, left ventricular ejection fraction; BMI, body mass index; BNP, B-type natriuretic peptide; MR, mitral regurgitation; TR, tricuspid regurgitation; LVESV, left ventricular end-systolic volume; LVEDD, left ventricular end-diastolic diameter; LAD, left atrial dimension; ARNi, angiotensin receptor-neprilysin inhibitors; ACEI, angiotensin-converting enzyme inhibitors; ARB, angiotensin receptor blockers; HBP, His-bundle pacing.*

### Lead Outcome of His-Purkinje Conduction System Pacing

There was a slight trend of increment in the correct threshold after follow-up in patients with HBCSP (from 2.13 ± 0.65 V/0.4 ms to 2.52 ± 0.42 V/0.4 ms, *P* = 0.051). The impedance decreased slightly after the follow-up (from 621.82 ± 135.80 Ω to 462.46 ± 109.95 Ω, *P* = 0.022). The correct threshold of the LBBB in patients with HPCSP was not different from that in patients with BiVP (2.13 V ± 0.65/0.4 ms vs. 2.36 V ± 0.45/0.4 ms, *P* = 0.351). All the changes are shown in Table 2. The pacing percentage at the final follow-up was 95.14 ± 4.17%.

**TABLE 2:** Changes in pacemaker parameters after HPCSP.

Parameters	During operation	Final follow-up	*P*-value
Capture threshold (V/0.4 ms)	1.87 ± 0.84	1.83 ± 0.96	0.895
Correct threshold (V/0.4 ms)	2.13 ± 0.65	2.52 ± 0.42	0.051
Impedance(Ω)	621.82 ± 135.80	462.46 ± 109.95	0.022

### Clinical Outcomes of His-Purkinje Conduction System Pacing

**FIGURE 1:**
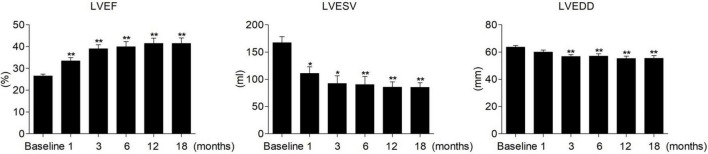
Continuous changes in LVEF, LVESV, and LVEDD after HPCSP. LVEF, left ventricular ejection fraction; LVESV, left ventricular end-systolic volume; LVEDD, left ventricular end-diastolic diameter. *vs baseline *P* < 0.05, **vs baseline *P* < 0.001.

Complete LV reverse remodeling was found in nearly half of the patients (45.24%) approximately 6.03 ± 3.50 months after the operation. Approximately 97.62% of patients responded to HPCSP. The LVEF value was higher than 50% in 23 patients (54.76%) soon after the operation (5.21 ± 3.10 months), and the LVEDD decreased to less than 50 mm in 21 patients (50.00%) approximately 6.84 ± 3.72 months after the operation. The changes in values such as QRS duration, cardiac structure, and cardiac function are shown in Table 3. The continuous changes in LVEF, LVESV, and LVEDD after HPCSP are shown in Figure 1.

**TABLE 3:** Changes in QRS duration, cardiac structure, and cardiac function.

	Baseline	Follow up	*P*-value
QRS duration (ms)	169.88 ± 19.17	113.67 ± 20.68	<0.001
LVEF (%)	26.52 ± 5.60	41.86 ± 11.56	<0.001
LVESV (ml)	167.67 ± 73.20	85.97 ± 62.24	<0.001
LVEDD (mm)	63.57 ± 8.19	55.46 ± 9.63	<0.001
LAD (mm)	44.59 ± 4.12	40.64 ± 4.68	<0.001
**MRgrade**			
Mild (n,%)	12(28.6)	20(47.6)	0.072
Moderate (n,%)	25(59.5)	18(42.9)	0.127
Severe (n,%)	5(11.9)	4(9.5)	0.724
**TR grade**			
Mild (n,%)	7(16.7)	23(54.8)	<0.001
Moderate (n,%)	23(54.8)	14(33.3)	0.048
Severe (n,%)	12(28.6)	5(11.9)	0.057
NYHA classification	3.31 ± 0.60	2.33 ± 0.75	<0.001

*LVEF, left ventricular ejection fraction; LVESV, left ventricular end-systolic volume; LVEDD, left ventricular end-diastolic diameter; LAD, left atrial dimension; MR, mitral regurgitation; TR, tricuspid regurgitation; NYHA, New York Heart Association.*

### Clinical Features of Patients With Left Ventricular Complete Reverse Remodeling

Univariate analysis showed that a short course of heart failure (*P* = 0.022), small LVESV before HPCSP (*P* = 0.008), and short QRS duration after pacing (*P* = 0.003) were related to LV complete reverse remodeling. Further multivariate regression analysis demonstrated that a short QRS duration was an independent predictor of normalized LVEF and LVEDD in patients with true LBBB and heart failure after HPCSP (OR 0.90, 95% CI: 0.84–0.97, *P* = 0.008), which is shown in Table 4. The area under the ROC curve was 0.819. The cutoff point was 107 ms with a sensitivity of 78.3% and a specificity of 77.9%.

**TABLE 4:** Predictors of LV complete reverse remodeling by univariate and multivariate analyses.

	Patients with LV complete reverse-remodeling (*n* = 19)	Patients with LV incomplete reverse-remodeling (*n* = 23)	Univariate			Multivariate		
			*P*-value	OR	95%CI	*P*-value	OR	95%CI
Male(n,%)	8(42.11)	14(60.87)	0.228					
Age(years)	68.95 ± 8.67	71.26 ± 9.68	0.414					
course of heart failure(years)	3.95 ± 2.89	6.32 ± 3.12	0.022	0.77	0.61–0.96	0.109	0.70	0.45–1.08
NYHA classification(level)	3.37 ± 0.50	3.26 ± 0.69	0.879					
LVEF(%)	27.58 ± 5.84	25.65 ± 5.37	0.267					
HBP (n,%)	(17, 89.47%)	(20, 86.96%)	0.670					
BMI(kg/m2)	25.24 ± 3.86	24.97 ± 3.20	0.810					
BNP(ng/L)	407.50(167.00, 2350.25)	541.00(230.00, 2683.50)	0.391					
Crea(μ mol/L)	71.00(60.21, 94.72)	82.00(62.31, 98.87)	0.697					
QRS duration(ms)	169.89 ± 16.47	169.87 ± 21.51	0.997					
QRS duration after HPCSP(ms)	102.21 ± 16.47	119.48 ± 21.73	0.003	0.94	0.90–0.98	0.008	0.90	0.84–0.97
MR grade			0.094					
Mild (n,%)	7(36.8)	5(21.7)						
Moderate (n,%)	11(57.9)	14(60.9)						
Severe (n,%)	1(5.3)	4(17.4)						
TR grade			0.717					
Mild (n,%)	3(15.8)	4(17.4)						
Moderate (n,%)	10(52.6)	13(56.5)						
Severe (n,%)	6(31.6)	6(26.1)						
LVESV(ml)	126.67 ± 51.38	201.83 ± 72.06	0.008	0.98	0.96–0.99	0.083	0.98	0.96–1.00
LVEDD(mm)	61.53 ± 7.40	65.26 ± 8.59	0.149					
LAD(mm)	43.39 ± 4.41	45.52 ± 3.70	0.109					
Diabetes mellitus (n,%)	7(36.84)	5(21.74)	0.285					
Hypertension (n,%)	11(57.89)	10(43.48)	0.354					
Chronic kidney disease (n,%)	1(5.26)	1(4.35)	1.000					
Coronary heart disease (n,%)	6(31.58)	8(34.78)	0.987					
Ventricular tachycardia/fibrillation (n,%)	3(15.79)	5(21.74)	0.626					
Atrial fibrillation (n,%)	3(15.79)	5(21.74)	0.243					
β -blockers (n,%)	18(94.74)	21(91.30)	0.915					
ARNI/ACEI/ARB	12(63.16)	14(60.87)	0.975					
Diuretics (n,%)	19(100.00)	21(91.30)	1.000					
Spirolactone (n,%)	19(100.00)	20(86.96)	0.999					
Statins (n,%)	11(57.89)	15(65.22)	0.496					
Aspirin (n,%)	4(21.05)	6(26.09)	0.644					
Nitrates (n,%)	6(31.58)	11(47.83)	0.236					

*NYHA, New York Heart Association; LVEF, left ventricular ejection fraction; BMI, body mass index; BNP, B-type natriuretic peptide; MR, mitral regurgitation; TR, tricuspid regurgitation; LVESV, left ventricular end-systolic volume; LVEDD, left ventricular end-diastolic diameter; LAD, left atrial dimension; ARNi, angiotensin receptor-neprilysin inhibitors; ACEI, angiotensin-converting enzyme inhibitors; ARB, angiotensin receptor blockers; HBP, His-bundle pacing.*

## Discussion

We proved that HBP and LBBP could dramatically improve heart function, and complete LV reverse remodeling was demonstrated in nearly half of the patients (45.24%) with true CLBBB and HFrEF. To the best of our knowledge, this was the first study to demonstrate that a short QRS duration after HPCSP was a strong independent predictor of LV complete reverse remodeling.

### Feasibility and Safety of His-Purkinje Conduction System Pacing

Although the report showed that the failure rate of BiVP was only 3.6%, it was unfortunate that the suboptimal position was accepted in approximately 20% of patients, which might impair the performance of CRT (12). We proved that the success rate of permanent HPCSP, including LBBP, reached approximately 90% in this study, which might be related to the combined application of HBP and LBBP (13, 14).

Complications such as thrombosis, infection, lead dislodgement and perforation, and other implant-related events were not found. Recently, Bhatt et al. reported that 8% of 101 patients with successful HBP implantation required electrode adjustment (15). In our study, the thresholds in most patients remained stable, with only two patients undergoing electrode adjustment 6 months after the operation. Consistent with our previous study, this study also demonstrated acceptable and stable thresholds for HBP 1 year after the operation (16).

The distal HBP lead helix, by virtue of being in the septal myocardium, played an important role in the favorable capture threshold and amplitude of the R wave (17). However, the failure of HBP was sometimes shown to be a non-negligible issue (18). For patients with a high threshold or failure of HBP, LBBP worked as a promising alternative for delivering physiological pacing to achieve electrical and mechanical synchrony.

### Clinical Performance After His-Purkinje Conduction System Pacing

Although BiVP was effective in reducing desynchronization, it was difficult to achieve complete reverse remodeling for the impaired conduction defect (19). This dilemma was somewhat circumvented with HPCSP (20). A series of publications suggested that HPCSP was a favorable choice for patients with CRT indications (21, 22). Li et al. reported that the response rate and super response rate in heart failure patients with LBBB were 88.9 and 44.4%, respectively, which were greater than those of BiVP (66.7 and 16.7%) (23). We showed that the response ratio was 97.62%, and the LV complete reverse remodeling ratio was 45.24% after HPCSP. For those patients with a CRT indication, would HPCSP be the best choice? We hope that an increasing number of studies will explore this issue in the future.

Huang et al. found that HBP obviously improved LVEF, LVESV, and NYHA classification in 74 patients with heart failure and LBBB (24). In our study, we also found that the LVESV, LVEDD, MR, and TR significantly improved after HPCSP. Furthermore, an improvement in LA remodeling after HPCSP was shown, which might predict the possibility of rhythm management in patients with atrial fibrillation during long-term follow-up.

The dramatic shortening of QRS duration after HPCSP was also demonstrated in our study (169.88 ± 19.17 ms vs. 113.67 ± 20.68 ms, *P* < 0.001). It has been proved that the shortening of QRS duration after HPCSP was more obvious compared to BiVP (mean QRS reduction of 20 ms) (25). But the shortening of QRS duration by LBBP was not as obvious as that by HBP (56 vs. 69 ms, *P* = 0.007) (26). It suggests that we should distinguish LV septal myocardial pacing (LVSP) from HPCSP due to its limited value on LV synchronization and physiological conduction system pacing (27). One of the differences is that LBBP can be fused with intrinsic RV activation for normal ventricular synchronization, whereas LVSP cannot.

### Patient Characteristics of Left Ventricular Complete Reverse Remodeling

Quite different from BiVP, HPCSP completely corrected the LBBB and resulted in super electrical resynchronization. This means that all heart failures originating from LBBB without other heart troubles would be cured. However, approximately 30% of the patients still suffered from heart failure, indicating that some other factor plays a role in LV reverse remodeling.

The course of heart failure was an important factor for LV reverse remodeling (28, 29). Similar to those studies, we also found that a longer course of heart failure was more common in patients with LV incomplete reverse remodeling, even though it was not an independent predictor in our study. This result suggests that the early correction of LBBB might be necessary to halt the progression of the cardiomyopathic process.

Current trials demonstrate that factors such as non-ischemic etiology, QRS duration, and morphology can predict BiVP response (30). It was also found that not all the cardiac complete reverse remodeling could be detected in patients with corrected LBBB in our study, which indicated that other etiologies might play a role in heart failure in one patient. Some patients’ conduction bundle lesions were not located at the proximal end of the trunk, which played a role in the normalized cardiac function. Some patients might be complicated by more myocardial lesions, and some patients might suffer from more scar burden.

One of the reasons for the failure of CRT *via* classical technology might be that too many “true LBBB” cases were contained, which did not meet the strict physiology-based criteria for “true LBBB” after all. It was reasonable to critically evaluate the definition of “true LBBB” and the physiology behind its ECG signature (31). However, QRS shortening plays a central role in the CRT response (32). In our study, we also proved that the short QRS duration after HPCSP was a strong independent predictor of LV complete reverse remodeling. The more synchrony there is after HPCSP, the higher the likelihood of a favorable outcome (33). Whether the difference between HBP and LBBP resulted in different QRS duration and cardiac functions will require further exploration in future studies. QRS duration and morphology reflect the electrical timing and activation sequence of the ventricles; thus, reversal of the electrical pathology indicates a potentially favorable effect of the therapy (34).

### Limitations

This was a single-center retrospective study with small sample size. More large-sample and randomized control multicenter studies might be necessary to confirm these results.

## Conclusion

His-Purkinje conduction system pacing dramatically reversed cardiac remodeling and cardiac function in patients suffering from HFrEF and true LBBB. To the best of our knowledge, this is the first study to prove that a short QRS duration after HPCSP is a powerful predictor of LV complete reverse remodeling after HPCSP.

## Data Availability Statement

The original contributions presented in the study are included in the article/supplementary material, further inquiries can be directed to the corresponding authors.

## Author Contributions

X-MG, D-NL, and F-LZ contributed to the conception and design of the study. F-LZ organized the database. D-NL, Y-NZ, and Y-HY performed the statistical analysis. B-LD and S-YD followed up with the patients. X-MG and D-NL wrote the first draft of the manuscript. L-JG, Y-XD, and Y-LX wrote the sections and proofread the manuscript. All authors contributed to manuscript revision and read and approved the submitted version.

## Conflict of Interest

The authors declare that the research was conducted in the absence of any commercial or financial relationships that could be construed as a potential conflict of interest.

## Publisher’s Note

All claims expressed in this article are solely those of the authors and do not necessarily represent those of their affiliated organizations, or those of the publisher, the editors and the reviewers. Any product that may be evaluated in this article, or claim that may be made by its manufacturer, is not guaranteed or endorsed by the publisher.
